# Ion-Specific Nutrient Management in Closed Systems: The Necessity for Ion-Selective Sensors in Terrestrial and Space-Based Agriculture and Water Management Systems

**DOI:** 10.3390/s121013349

**Published:** 2012-10-01

**Authors:** Matthew Bamsey, Thomas Graham, Cody Thompson, Alain Berinstain, Alan Scott, Michael Dixon

**Affiliations:** 1 Canadian Space Agency, Space Science and Technology, 6767 route de l’aéroport, Longueuil, QC J3Y 8Y9, Canada; E-Mails: mbamsey@uoguelph.ca (M.B.); alain.berinstain@asc-csa.gc.ca (A.B.); 2 Controlled Environment Systems Research Facility, School of Environmental Sciences, University of Guelph, 50 Stone Road East, Guelph, ON N1G 2W1, Canada; E-Mails: tgraham@uoguelph.ca (T.G.); cthompso@uoguelph.ca (C.T.); 3 COM DEV Ltd., 303 Terry Fox Dr., Suite 100, Ottawa, ON K2K 3J1, Canada; E-Mail: alan.scott@comdev.ca

**Keywords:** ion-selective sensors, water quality, inorganic ion monitoring, space exploration, bioregenerative life support, hydroponics

## Abstract

The ability to monitor and control plant nutrient ions in fertigation solutions, on an ion-specific basis, is critical to the future of controlled environment agriculture crop production, be it in traditional terrestrial settings (e.g., greenhouse crop production) or as a component of bioregenerative life support systems for long duration space exploration. Several technologies are currently available that can provide the required measurement of ion-specific activities in solution. The greenhouse sector has invested in research examining the potential of a number of these technologies to meet the industry's demanding requirements, and although no ideal solution yet exists for on-line measurement, growers do utilize technologies such as high-performance liquid chromatography to provide off-line measurements. An analogous situation exists on the International Space Station where, technological solutions are sought, but currently on-orbit water quality monitoring is considerably restricted. This paper examines the specific advantages that on-line ion-selective sensors could provide to plant production systems both terrestrially and when utilized in space-based biological life support systems and how similar technologies could be applied to nominal on-orbit water quality monitoring. A historical development and technical review of the various ion-selective monitoring technologies is provided.

## 1. Introduction

**Figure 1.**
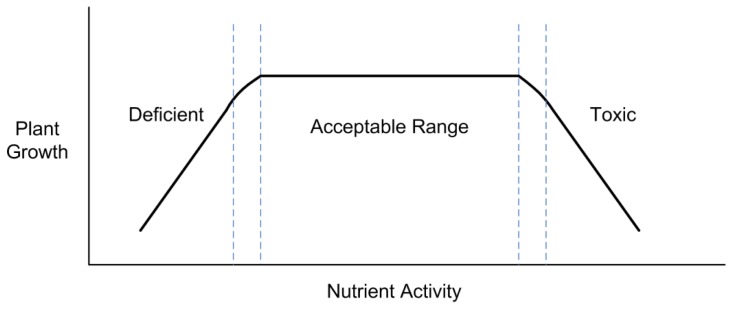
General principle of plant nutrient acceptable/sufficiency ranges. Dashed vertical lines represent marginal zones between acceptable and deficient or toxic nutrient activity.

Plants require a wide range of nutrients to support their growth, development, and reproduction. Each of these specific nutrient ion species has an ionic activity* window within which growth is optimized. *Activity*, although less commonly used by the greenhouse industry, is related to concentration and is in fact the more important fundamental parameter with respect to plant nutrition (Mengel as cited in [1]). Additionally, it is ion activity, not concentration which ion-selective sensors typically measure. The distinction between activity and concentration is more fully elaborated in Section 1.1. The caveat to this is that all the nutrient ion species need to be within their respective activity windows if plant productivity is to be optimized. Departure from these optimal levels in any of the nutrient ions will have an influence on all the others as well. The uptake and utilization of nutrients depends not only on the absolute quantities (in soil or solution), but also on the ratios among nutrient species. Deviations above or below these ion activity regimes can lead to the development of toxicity or deficiency symptoms and ultimately impair productivity. These acceptable nutrient ion activity ranges, which are often termed sufficiency ranges, can be visualized for a given nutrient ion in Figure 1.

Many greenhouse operators now employ hydroponics, where plants are grown in a soilless or inert support substrate with the vast majority of nutritional requirements supplied via the irrigation water. Hydroponic crop production systems allow for much tighter control over nutrient inputs (cf. soil based systems). When nutrients are supplied in this fashion, the term fertigation (fertilizer and irrigation) is used in place of, or interchangeably with, irrigation. Providing nutrients at their optimal levels allows growers to achieve maximum yields and can conserve both water and nutrients compared to other plant production systems [2]. Traditionally, the nutritional appropriateness of hydroponic fertigation solutions used in greenhouse plant production is obtained by monitoring solution pH and electrical conductivity (EC) [3,4]. Although this provides some information about the nutrient ions present in the solution, EC is an indiscriminate measure for the total nutrient composition and does not differentiate among the nutrient species present [5,6]. Likewise, any non-nutrient ions within the solution will also contribute to the solution EC, and thus if present, these ions can result in non-ideal nutrient management choices. Further, different nutrient ions contribute disproportionately to the measured EC value, which can skew the interpretations made based on the EC metric (e.g., although potassium and sodium ions have the same charge, potassium contributes more strongly, for an equivalent increase in activity, to solution EC [7]). Moreover, in addition to the indirect effect pH has on EC by inducing precipitation/dissolution reactions, as H^+^ and OH^−^ contribute differently to solution EC, pH changes can influence EC measurements, further complicating the utilization of EC as a tool for assessing nutrient status [8].

Plant productivity can be influenced by the activity of any one nutrient ion species. Given this, pH and EC measurements alone do not provide sufficient information to allow growers to realize optimal plant production from a solution fertility perspective. Some greenhouse growers do attempt to manage their nutrient solutions based on individual nutrient species, however their efforts are often temporally restricted. Currently growers are limited to relatively infrequent (e.g., 1–3 weeks) off-line analysis in which nutrient solution samples are physically mailed to off-site accredited laboratories [9]. During the shipping, processing, and reporting lag time, the status of the on-farm nutrient solution will have changed, potentially to a significant degree, limiting the usefulness of what is often expensive data [10]. Plant tissue/leaf analysis serves as an additional off-line analysis technique informing adjustment decisions but also presents the disadvantage of cost and time lag between sampling and result delivery [2,11].

**Figure 2.**
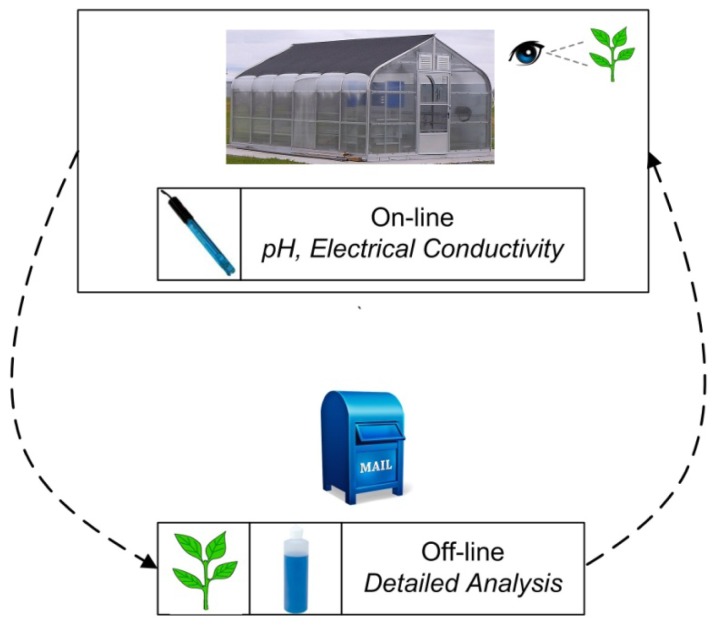
Conventional greenhouse nutrient solution monitoring. On-line monitoring of pH and EC is typically conducted and supplemented with grower observations of plant visual symptoms. Off-line analysis of plant tissue or samples of the hydroponic nutrient solution is also conducted at various intervals by sending samples off-site to accredited laboratories. Some or all of this information is utilized to make nutrient solution adjustment decisions well after the initial sampling period.

Many growers rely on experience and a keen eye to detect (visually) the symptoms of nutrient deficiency and/or toxicity. Although effective, this method is reactionary in nature. In most cases, visual symptoms are manifested only after prolonged periods of growth in a non-optimal nutritional environment. In certain cases, visual symptoms for different deficiencies/toxicities can be very similar, resulting in a misdiagnosis and potentially leading to inappropriate solution modifications that can further exacerbate the problem [12]. Figure 2 provides a graphical overview of the conventional means that greenhouse operators employ to obtain information used to make nutrient solution and application adjustment decisions.

The capacity to achieve high fidelity feedback control of the nutrient solution composition will only be possible with robust, on-line, ion-selective sensors. Unfortunately, although these sensors have been much sought after, they have not yet found widespread use and there remains a technology and application gap between the needs of the greenhouse industry and available ion-selective sensing technologies [13–15].

Beyond the obvious terrestrial needs, on-line ion-selective sensors are considered to be a requirement for plant production in biological life support systems, which many consider to be a critical component to long duration human space exploration [16,17]. As will be described, similar inorganic sensing systems are also highly required for water quality monitoring in on-orbit recovery and treatment systems both currently aboard the International Space Station (ISS) and for extended duration missions to other solar system destinations.

### Concentration vs. Activity

Although more commonly utilized and understood, concentration of a nutrient ion within the hydroponic solution is not the most fundamental variable related to the definition of nutrient ion sufficiency ranges. In fact, although related to concentration, it is nutrient ion activity for which growers and operators should be most familiar with, as it is this variable which directly influences plant growth and which ion-selective sensors will typically measure (Mengel as cited in [1,18]). As it is typically less understood by the greenhouse industry at large, a description and mathematical distinction between ionic activity and concentration are provided. Activity is the “effective concentration” of a given species in a solution. It can be related to concentration via Equation (1).

(1) a=γc

where a is the activity (M), *γ* is the activity coefficient (unitless) and c is the concentration (M) of the species in question. In an electrolyte solution the activity coefficient of a given ion is not accessible because it is experimentally impossible to independently measure the electrochemical potential of a given ion because the solution contains both positively and negatively charged ions. Instead a mean activity coefficient is defined. This mean activity coefficient (*γ*_i_) is usually estimated using the Debye-Hückel formula or some variation thereof [19,20]. The basic Debye-Hückel formula is presented in Equation (2) [21]:

(2) $$-\log\gamma_{i}=\frac{Az^{2}\sqrt{I}}{\left(1+B\propto \sqrt{I}\right)}$$

where *γ*_i_ is the mean activity coefficient, *I* is the ionic strength (M), z is the charge number of the ion, A and B are temperature dependent constants and α is an ion size parameter (nm). If assuming an aqueous solution and a temperature of 25 °C the expression simplifies to:

(3) $$-\log\gamma_{i}=\frac{-0.509z^{2}\sqrt{I}}{1+(3.29\propto \sqrt{I})}$$

For non-aqueous solutions or solutions which differ significantly from 25 °C, the coefficients A and B differ from those listed in Equation (3) and can be calculated separately [22]. Tabularized values for A and B can also be obtained in the literature [23]. The Debye-Hückel method is an accurate model when considering dilute solutions but becomes less accurate as the considered solution becomes less dilute. Typically the basic Debye-Hückel method is good for solution ionic strengths up to approximately 0.1 M while an extended Debye-Hückel method (e.g., the Davies equation) can be utilized for solutions up to approximately 0.5 M [20]. Other methods have been proposed for more concentrated solutions [24]. As seen from the equations above, in dilute solutions (<10^−4^ M) the activity coefficient has a value close to unity and in these instances, activity approaches concentration. Oppositely, as ionic strength increases, the activity coefficient decreases and thus results in a greater difference between the concentration and activity.

**Figure 3.**
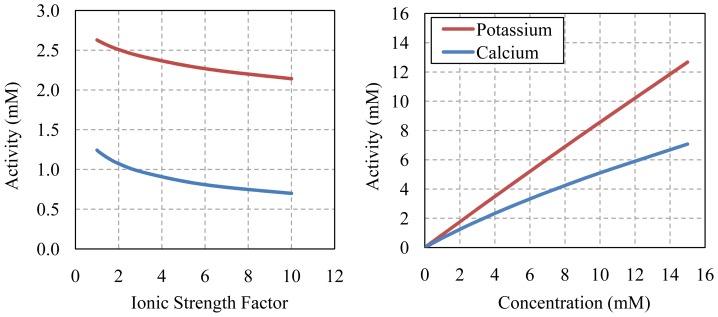
Examples of the distinction between activity and concentration for the monovalent ion potassium and divalent ion calcium within half strength Hoagland nutrient solution. (**Left**) Ionic activity changes for the fixed ion concentrations of potassium ([K^+^] = 3 mM) and calcium ([Ca^2+^] = 2 mM) in half strength Hoagland nutrient solution for changing ionic strength. The ionic strength factor is a ratio of the ionic strength of the solution compared with the ionic strength of the nominal half strength Hoagland nutrient solution. (**Right**) Activity *versus* concentration of potassium and calcium for nominal half strength Hoagland nutrient solution.

Figure 3 visually presents examples of how ion activity varies with ionic strength and subsequently with concentration for the two differently charged nutrient ions, potassium and calcium, for the nominal half strength Hoagland nutrient solution at 25 °C (*i.e.*, I ≈ 0.0162 M, [K^+^] = 3 mM, [Ca^2+^] = 2 mM).

The activity coefficient is thus a factor used in thermodynamics that aids in accounting for deviations between the ideal behaviour of a mixture of chemical substances and the actual behaviour. In general, activity depends on any factor that alters the chemical potential of a solution. These factors can include among others; temperature, pressure, electric fields, magnetic fields as well as the composition of the mixture itself (*i.e.*, absolute amounts of the constituents), the latter being the most often considered account of activity.

## 2. Justification for the Development of On-Line Ion-Selective Sensors

Reliable, on-line ion-selective monitoring could provide terrestrial greenhouse growers and research scientists with the following:

- Increased crop growth yields
- Improved fruit/crop quality
- Improved crop growth reliability
- Reduced fertilizer use
- Reduced water use
- Reduced water and nutrient discharge (environmental compliance)
- Improved nutrient control system reliability
- Reduced susceptibility to source water variation
- Method to test plant growth media
- Decreased labour requirements
- Enhanced scientific knowledge of plant biology and plant-environment interactions

These benefits can be separated into crop specific and system level benefits to the grower and are described herein.

### 2.1. Crop Requirements

**Figure 4.**
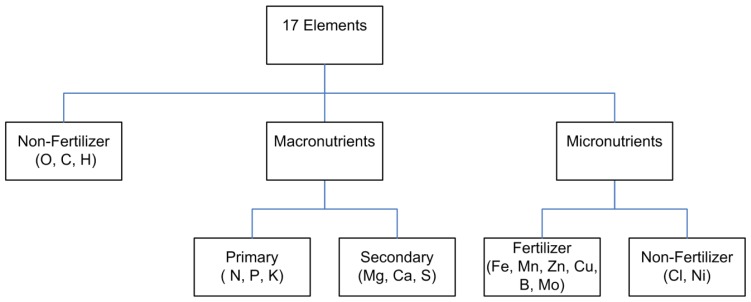
Categories of essential plant nutrients.

The essentiality of an element is determined through the exclusion of the suspected nutrient and observations on the physiological impacts of that exclusion. If the plant cannot survive in the complete absence of the element then it is by definition an essential plant nutrient. There is still debate regarding the essentiality of some micronutrients as the amounts required for plant survival are extremely low (e.g., nickel) and it can be exceedingly difficult to exclude them with full confidence. As such, there are anywhere from 15 to 18 reported essential plant nutrients that govern plant growth, development and reproduction [2,25,26]. Three elements, oxygen, carbon and hydrogen, can be considered non-mineral or non-fertilizer nutrients as these are obtained directly through CO_2_ and H_2_O via photosynthesis. In comparison, the remaining essential elements are obtained from the soil, the fertigation solution, or through atmospheric deposition leading to foliar uptake. Nutrients can also be classified according to the quantities required by the plant with those required in relatively large quantities termed macronutrients and those required in lesser quantities termed micronutrients as portrayed in Figure 4.

Plant nutrients occur in different forms within the nutrient solution. Many occur as monatomic ions (e.g., K^+^, Ca^2+^, Zn^2+^, Cl^−^, *etc.*), while others occur as polyatomic ions (e.g., NH_4_^+^, NO_3_^−^, HPO_4_^2−^, H_2_PO_4_^−^). Each particular nutrient has its own effects on the plant and their individual activities can have a very strong influence on plant growth and health. If a particular nutrient is deficient, plant yields can be negatively affected [4,27–29]. A similar reduction in plant growth can arise when a particular nutrient is present at a concentration that is too high. This can lead to direct toxic effects, but can also limit or alter the uptake of other nutrient ion species. This interference pattern can potentially result in a situation where there are both toxic and deficiency symptoms generated by the imbalance of a single nutrient species [2,30]. If the activity of each plant nutrient is maintained within their specific sufficiency ranges (and other environment variables are acceptable) then optimum plant growth will be obtained. Although micronutrients are required in lesser amounts than macronutrients, each nutrient has its own sufficiency range and micronutrient deficiencies or toxicities can be just as devastating as macronutrient deficiencies or toxicities.

It is clear that managing plant nutrient requirements can become very complex, particularly when dealing with closed or recirculating systems. This being said, acceptable activity ranges can be defined for each particular plant nutrient ion for a given plant species. Knowledge of these acceptable ranges, combined with a sensor and nutrient control system capable of monitoring and responding to crop needs in an ion-specific manner could readily ensure reliable and optimized production even in high density cropping systems (*i.e.*, greenhouse and bioregenerative life support systems).

Further reasoning for the criticality of ion-selective sensors includes:

- Nutrient requirements vary among crops [2,27,29,30]
- Nutrient requirements change over the lifetime/growth phase of a given plant [4,30–35]
- Nutrient requirements fluctuate with changing environmental conditions [30,33,36]
- There exist several key nutrient ion ratios that must be maintained within the nutrient solution [29,30].

As a manner to demonstrate these statements, examples of each are taken for the nutrient ion potassium:

#### 2.1.1. Variation among Crops

Requirements for potassium vary among crop species [37,38]. As an example, compare typical nutrient solution recipes for tomato and pepper. Tomato fertigation solutions, on average, call for about 6 mM total potassium concentration, while those for pepper are up to 50% higher at 9 mM total potassium (assumes identical plant growth system and nutrient solution application rate) [2]. Furthermore, fruiting crops typically have higher potassium requirements than do vegetative crops [2].

#### 2.1.2. Growth Phase

Potassium is a particularly notable example of a nutrient ion in which plant requirements change considerably across developmental stages, especially during fruiting stages [39]. Seedlings and young plants typically require lower levels of potassium while the requirements increase with fruit load [10,35]. For example, work suggests tomato potassium concentration requirements of 120 ppm (∼3.1 mM) after transplanting with levels increasing to 200 ppm (∼5.1 mM) during fruiting [34]. In fact, as the predominant cation in tomato fruit, potassium has major effects on fruit quality and development, explaining why it must be maintained at higher activities during fruiting stages than during vegetative stages [35].

#### 2.1.3. Environment

From the perspective of environmental conditions, both the aerial and root zone environments can drive specific nutrient uptake. For example, in a study of hydroponically grown tomatoes it was demonstrated that the uptake of nitrogen, potassium and calcium were primarily a function of solar radiation and air temperature, while phosphorus uptake was influenced more strongly by root temperature [38,40]. As the use of complex environment-plant models to predict this environmentally influenced selective nutrient uptake is beyond the scope of current knowledge, on-line nutrient solution sensors are required to provide real-time interpretation of these environment effects [39].

#### 2.1.4. Nutrient Ratios

Monitoring and controlling fertility levels based on individual ion activities ensures that control is maintained over plant-nutrient and nutrient-nutrient interactions. High concentrations of a given plant nutrient can interfere with the availability and uptake of other nutrients (antagonism) or can increase the demand for a different nutrient ion (stimulation) [26]. High potassium activities for instance can suppress magnesium and/or calcium uptake. This uptake suppression can be sufficiently large to induce magnesium and/or calcium deficiency symptoms even if solution activities of these two nutrients are within their optimal range. In the absence of ion-specific data, managers would treat the situation with further additions of the ‘deficient’ nutrient. This remedy is wasteful and can lead to additional nutritional issues, ultimately leading to the need to discard the nutrient solution [26,29]. Similarly, high magnesium or calcium can induce potassium deficiencies, leading to the same scenario that ends with the discarding of the solution [2,29]. The perception may be that these problems only arise after prolonged nutrient solution recycling; this is not the case. Research indicates that even growers who use conventional sensing and management practises with frequent solution changes (e.g., weekly) may still experience suppressed crop productivity resulting from nutrient ion imbalances [2].

### 2.2. System Level Benefits

Additional rationale for on-line ion-selective sensor technology stems from the observed benefits it provides at the overall plant growth system level.

#### 2.2.1. Improving Fertilizer and Water Use Efficiency

Ion-selective sensing can improve the efficiency of nutrient use within a plant growth system [41–43]. A more complete understanding of nutrient solution dynamics ensures that nutrients are replenished at a rate more closely tracking uptake by the plant. In traditional sensing schemes, where no ion-selective sensing is available, a low EC measurement will typically result in the addition of a suite of nutrients where in actuality only one nutrient activity may be below nominal. This fertilizer addition will thus result in certain nutrients being added without being required and thus wasted. As the acids and bases typically used by growers to maintain pH within desired bounds are salts that include nutrient ions (e.g., HCl, HNO_3_, NaOH), adjusting pH results in the activity of another nutrient ion being increased, which if not understood can influence the overall system.

Ion-selective sensing will also ensure efficient use of water as tighter controls on its applicability for plant growth will better improve the capacity for nutrient solution recirculation over longer periods. Presently, many growers flush their nutrient solution when they do not have full confidence in its quality, usually based on a predetermined EC discharge criterion [13]. Improved access to nutrient status data will reduce the need for the flushing of effluent and thus reduce nutrient and wastewater outputs [43,44]. Studies directly comparing open *versus* closed systems for various crop types have shown savings in both water and nutrients for equivalent plant yields in closed systems [45]. Additional studies have shown that closed Dutch greenhouses saved up to 30% water and 40% fertilizers compared to traditional open systems [43,46]. Full closure of Dutch greenhouses is limited by the relatively poor quality of supply water, primarily due to elevated sodium levels [46]. Canadian data has suggested that greenhouse tomato production in rockwool leached approximately 7.5 tonnes of fertilizer salts per year for every hectare of production area [47]. The same dataset suggested that 4,000 m^3^ of leached irrigation water was produced for the same one hectare production area while studies from semi-arid regions suggested, that open irrigation systems produced approximately 10,000 m^3^ of discharged irrigation water per hectare of greenhouse production [13,47].

#### 2.2.2. Environmental Compliance

Water shortages and contamination have become a major concern for governments and other stakeholders. In response, governments around the world are enacting legislation aimed at conserving and protecting water resources [3,46,48–52]. Globally, agriculture is the single largest user of freshwater resources, accounting for approximately 70% of all freshwater abstraction [53,54]. Increasing restrictions on freshwater availability necessitates the need for ion-selective sensors to allow for more intensive and efficient water recycling and nutrient management.

The European Union Water Framework (EUWF) obliges member states to achieve fixed output levels in all areas of agricultural activity, including effluent restrictions on nitrate and phosphate per unit growing area, by 2027 [55]. In fact, greenhouse growers in countries such as the Netherlands were required by law to re-use their irrigation water on a zero discharge basis starting in the year 2000 [56]. The regulations were relaxed when it was realized that the technology did not yet exist to adequately control a recirculating irrigation solution in perpetuity, but the goal remains. In Asia, the Chinese government has also introduced water restrictions for agricultural production, requiring a 20% reduction in water use by the year 2020 [52]. After severe droughts in the 1990's, the United Kingdom introduced the Water Act (2003), which requires strict licencing and demonstration of efficient irrigation water management when applying for water taking permits [57]. The United States also has legislation that requires the treatment and/or recycling of wastewater originating from point source municipal and industrial operations. Several states (e.g., California, Delaware, Florida, Maryland, Michigan, North Carolina, Oregon, Texas) have included greenhouse and nursery growers as industrial point sources under their legislation [58,59].

The province of Ontario (Canada), in the events surrounding the municipal water contamination in Walkerton, Ontario, implemented sweeping law and policy changes including the 2002 enactment of the Sustainable Water and Sewage Systems Act [60], the Safe Drinking Water Act [61] and the Nutrient Management Act [51]. Although not formally implemented until afterwards, the general strategy and draft bylaws of the Nutrient Management Act had been in development since as early as 1997 [62]. These acts, as well as other provincial acts including the Environmental Protection Act of 1990 [63] directly affect the management of nutrients on farms and in greenhouses. The Nutrient Management Act has the explicit purpose to “provide for the management of materials containing nutrients in ways that will enhance protection of the natural environment and provide a sustainable future for agricultural operations and rural development” [51]. In addition to providing requirements for documentation and tracking of nutrient applications, the act sets compliance measures [51]. At the time of submission, greenhouses did not fall under the Ontario Nutrient Management Act but discussions are underway for their inclusion [64]. Nevertheless, greenhouses like any other entity must still comply with the Ontario Environmental Protection Act regulations [63]. Adding to the complicated regulatory framework, other federal regulations then overlay on the provincial acts such as the Canadian Environmental Protection Act [65] and Canada's Fisheries Act [66].

#### 2.2.3. Improved Nutrient Control System Reliability

Monitoring ion-selective nutrient activities and their individual control will have benefit from the perspective of reducing the probability of plant growth hardware failures. For example, if particular ion activities become excessive, precipitates could form causing damage or blockage in certain components (e.g., injectors, tubing, *etc.*) [67]. A possible and often reported example within hydroponic plant growth systems is the clogging of emitters due to the formation of gypsum (CaSO_4_) precipitate when elevated calcium and sulphate concentrations are reached [13]. In addition, nutrient and water delivery system components can change over time due to regular wear or failure, producing changes in either the amount of water delivery or the amount of nutrient, acid or base injected. Ion-selective monitoring would help growers better identify the potential hardware problem by providing the capacity to note that a given activity did not change as much as would have been expected when performing control activities on the nutrient solution.

#### 2.2.4. Accounting for Source Water Variation

Sources and quality of irrigation water can vary considerably depending, primarily, on climatic and geologic conditions [52,68–72]. Sources of irrigation water can be split into two general categories: (1) surface water sources, and (2) groundwater sources. Surface water sources comprise of rivers and streams (flowing sources), lakes and reservoirs (dams), stream and run-off fed ponds and catch basins, rainwater collection (cisterns and ponds), and recaptured irrigation water. Groundwater sources are primarily well water, but spring-fed ponds/reservoirs can also be included in this category. Municipal water is also used as an irrigation supply and would be categorized based on the source that the municipality draws from.

The quality of an irrigation water source is highly variable, particularly for surface water [73,74]. Surface water is more mutable as it is directly influenced by a greater number of factors, including but not limited to precipitation events, evaporation, surface run-off (contamination), and biotic modification (flora and fauna within the system) [73–75]. Groundwater tends to be qualitatively stable at a given site, but can vary significantly between sites [75].

Irrigation source water quality can be characterized based on several key chemical parameters that directly or indirectly influence plant productivity. Direct effects in the form of toxicity and/or deficiency can occur, while indirect effects can result from chemical interference with nutrient ion uptake or availability [72]. The primary parameters of concern are: (1) pH; (2) alkalinity (carbonate/bicarbonate levels); (3) hardness (calcium and magnesium); (4) total salinity (measured as electrical conductivity); (5) ionic composition; and (6) pathogens (human and plant) [72,75,76]. Typical or desirable ranges for raw irrigation water are presented in Table 1.

##### pH and Alkalinity

Knowledge of irrigation source water pH is critical for crop production applications. The pH of a solution has a significant influence on nutrient availability, regardless of the absolute amount of a nutrient in solution. The effect is particularly evident when considering transition metal micronutrients (Figure 4) which are largely unavailable to plants at pH > 7.0. At pH < 5.0 the same absolute amounts of these micronutrients can become toxic as their bio-availability (to plants) is increased significantly.

Closely tied to pH is alkalinity of the solution. Alkalinity is the quantitative capacity of a solution to neutralize acid, largely through the action of carbonates (CO_3_^2−^) and bicarbonates (HCO_3_^−^), and is expressed as milliequivalents of calcium carbonate (meq·L^−1^ CaCO_3_; 1 meq·L^−1^ CaCO_3_ = 50 mg·L^−1^ CaCO_3_) [72,76]. Solutions high in alkalinity tend to raise the pH of the growth substrate, with repeated applications, through the scavenging of H^+^ ions in the media [72]. The elevated root media pH can lead to deficiency symptoms, and the high buffering capacity of the solution can make it difficult to lower the pH once it has been elevated.

##### Hardness, Total Salinity, and Ionic Composition

The ionic composition of irrigation source water plays a critical role in the development of an overall nutritional management plan. The background levels of nutrient and other influential inorganic ions need to be considered when developing and maintaining a nutritional plan for a crop.

Calcium and magnesium can naturally occur at high levels when the source water is extracted from aquifers in calcite and dolomite formations (hard water). Typical nutrient solution concentrations for calcium and magnesium (pepper, tomato and lettuce) are approximately 200 mg·L^−1^ and 70 mg·L^−1^ respectively [77]. In irrigation water drawn from hard water aquifers, the background calcium and magnesium levels can represent a significant portion of the recommended application. As such, it is critical that this background contribution be accounted for in the nutritional plan for the crop [72].

Potassium, phosphate, ammonium, nitrate and sulphur are not typically present at levels that will dramatically influence the overall nutritional status of the solution unless there has been a contamination of the water source from fertilizer or other sources [72]. This said, contamination can occur with surprising frequency [78] and it is important both from an environmental and crop production perspective to monitor irrigation source water for these ionic species.

Sodium, chloride, and fluoride can also occur at high levels, particularly in municipal water sources [72]. These ions can interfere with the uptake of calcium and promote leaching of calcium and magnesium from the growth substrate [72]. In addition to these interference effects, direct toxicity can also occur (e.g., leaf burn).

##### Pathogens

Pathogens, both human and plant-based, are a major concern when considering the suitability of a water source for irrigation. Although pathogen analysis is beyond the scope of this review, it is worth noting that recent developments in molecular biology have led to the first generation of on-site, rapid, low technical requirement pathogen identification systems [79–81]. Although much work still needs to be done, on-line analysis of pathogens in an irrigation solution should soon be a reality.

#### 2.2.5. Method to Test Plant Growth Media

As growth substrates themselves can absorb and leach material into the nutrient solution they can have an influence on plant growth if these fluxes cannot be monitored. The fact that various types of growth media (polymer, coconut coir, mineral wool, *etc.*) exist and that the same product can vary among manufacturers implies even more variability in growth conditions [82]. On-line ion-selective sensors will ensure that the growth substrate variability can be adjusted by active control of the nutrient solution. These sensors will also benefit already established media analysis programs (e.g., 1:2 dilution, 1:5 dilution, saturated media extract and pour through procedure) whereby media is tested before and after planting by running water through it and testing the leachate. As the solution contained in these media is the primary source of nutrients for plant growth, the extracted water gives a good indication of the available nutrient status.

In a general sense, ion-selective monitoring of the bulk nutrient solution and the manipulations to the ionic make-up based on that monitoring, need to be combined with an understanding of the transport of nutrients to the roots within the growth medium. The transport of nutrients through the growth substrate and to roots is governed by mass flow and diffusive processes that will dictate the effectiveness of any solution management system [83]. Ultimately, the control system will require specific ion concentration data, which would feed into a mass flux driven control algorithm that takes into account the effects of growth medium capacitance and plant uptake [83]. This combined system of ion-specific data and mass transport modelling would ensure sufficient nutrient delivery to satisfy demand at the root surface while avoiding potential damage due to root surface fertilizer accumulation.

#### 2.2.6. Decreased Labour Requirements

Labour is one of the largest costs to a Canadian greenhouse grower, totalling approximately 28.5% of total greenhouse operating expenses, therefore any reduction in labour requirements can be of benefit [84]. Although not yet conclusive, researchers have suggested that due to their ability to provide an improved understanding of crop nutrient uptake, ion-selective sensors can increase automation and thus reduce human tending requirements [41,85].

#### 2.2.7. Other Benefits

Continuous use of UV, ozone, or chlorine for pathogen control in the nutrient solution are known to result in changes in nutrient solution chemistry which if not detected and adjusted for, can impact plant yields [86]. The aforementioned needs for ion-selective sensing are in addition to the benefits that ion-selective nutrient solution management would provide to growers in terms of increased shelf life [30], taste manipulation [30] and improved food safety [33,42,87].

### 2.3. Ion-Selective Sensors as a Tool for Plant Scientists

Many of the advantages of ion-selective sensors stem from the increased understanding of the growth environment that they provide along with concurrent assessments of plant physiological responses. Their application to scientific studies can further the understanding of plant growth and physiology by providing high fidelity control and understanding of the nutritional environment, allowing scientists to isolate effects often lost in the noise of crude monitoring methods such as EC [6].

It seems obvious that a better understanding of nutrient uptake in relation to other environmental conditions, especially light, will yield significant scientific advances. Plant science has long appreciated the influence of various light spectra on plant growth and development [88]. A number of studies have assessed the relationship between light quality and uptake of mineral nutrition in a variety of plant species [89–91]. Others, using special filters, investigated the effects of blue and red light on nitrate reductase levels in maize and pea and showed that the efficacy of blue light was dependent on the concentration of nitrate in the nutrient solution [92]. Ion-selective sensor technology would clearly be a boon to the interpretation of plant-environment interactions such as these.

Plants are constantly modifying the physical and chemical characteristics of their root environment through the exudation of a wide range of chemical compounds [93]. Such chemically and functionally diverse compounds include, chelates, reducing compounds, and hydrogen for soil acidification [94,95]. Ion-selective sensors will allow the study of several of these processes and their influence on the nutrient solution. Further benefit would result when this information is available in real-time.

### 2.4. Space-Based Biological Life Support Systems

As astronauts venture farther and stay for extended periods away from Earth, material recycling (*i.e.*, closure of their air, water and food loops) becomes ever more necessary. Biological life support systems for human spaceflight have been proposed and studied for some time [96,97]. Several excellent reviews of the history of bioregenerative life support systems exist [98–101]. Their benefit stems from their ability to provide edible biomass production, carbon dioxide absorption, oxygen generation, water recycling and waste degradation [102,103]. Evidence also suggests that the use of higher plants could provide the crew with psychological benefits on long duration space missions [104,105]. There is general consensus in the community that bioregenerative life support systems become more advantageous in comparison to more traditional physical-chemical based life support systems for distant, long duration missions where resupply becomes too costly [106–111]. This is particularly true as biological systems are presently the only feasible way to generate food [103,112,113].

Canada has been active in the biological life support domain since the early 1990s [114]. Nutrient management has been a key research area in this work and investigations of several ion-selective sensor types having been conducted [14,115,116]. In recent years, Canadian researchers have developed the Canadian Advanced Life Support Systems (CanALSS) Roadmap [114,117,118]. The CanALSS Roadmap has a very strong focus on sensor development for plant growth systems, with ion-selective sensors clearly targeted as a Canadian priority. Several technology roadmaps and research groups in various countries have also defined ion-specific sensors as a key technology/priority for bioregenerative life support systems and elaborated on their specific benefits [16,119–121]. Space-based plant production systems supporting human crews must achieve high yields, produce minimal waste and have high reliabilities. Such high closure systems will only be possible with an on-line knowledge of the hydroponic nutrient solution, thus ion-selective sensors.

## 3. Ion-Selective Sensor Technologies for Terrestrial and Space-Based Plant Production Systems

Numerous sensor technologies exist for ion-selective sensing in solution. Although this is a constantly developing field of research, with new sensors developed on a constant basis, this section presents a basic functional description and development history of several of the currently applied technologies. While there is reasonable variance in the extent to which even the presented technologies have been investigated, each sensor has certain advantages and disadvantages with respect to other sensor types. Following a description of each technology, a basic metric based summary assessment of the respective sensing technologies is presented.

### 3.1. High-Performance Liquid Chromatography (HPLC)

High-performance liquid chromatography (HPLC), and more recently ultra-high-performance liquid chromatography (UHPLC), is the standard analytical system for the separation of components in a liquid matrix [122,123]. When discussing the separation of ionic species, HPLC is further classified as ion chromatography (IC). Although HPLC/IC can be used for the quantification of a wide range of compounds, discussion here will be limited to methods and systems used in the quantification of inorganic anions and cations comprising plant nutrients (Figure 4). There are two main processes that constitute IC analysis: (1) separation of the analyte ion(s) of interest; and (2) detection and quantification of the analyte(s) of interest. The following details the processes relevant to plant nutrient ions.

#### 3.1.1. Separation Methods

Although there are four separation methods commonly associated with IC (ion-exchange, ion exclusion, ion pairing and reverse-phase), only ion-exchange is in routine use for plant nutrient ion analysis. As the name implies, separation is based on an ion-exchange process that occurs between the mobile phase and the permanently charged ion-exchange groups bonded to the stationary phase. In anion nutrient separation these exchange groups are quaternary ammonium, while for cations sulfonate groups are employed [122,124]. The counter ions for these functional groups are in close physical proximity, which allows the system to maintain electrical neutrality [122]. Analyte ions are temporarily exchanged with the counter ions; retained by the fixed charge of the stationary phase. The analyte ions are retained for varying lengths of time based on the affinity (electrostatic forces) that the analyte has towards the stationary phase, thereby allowing separation of the constituent ions [122,124].

#### 3.1.2. IC Detection Methods

##### Electrochemical Detection

Electrical conductivity detection is the most common detection method for plant macronutrient ions (e.g., NO_3_^−^ and Ca^2+^), and is based on changes in conductivity of the mobile phase due to the presence of an analyte. The mobile phase has a stable conductivity under a given set of conditions (temperature, pressure, flow rate, *etc.*) representing the baseline conductivity for the analysis. When an analyte is remobilized from the stationary phase, it changes the overall conductivity of the mobile phase passing through the detector. That change is recorded as an increase or decrease in the baseline conductivity, depending on the mobile phase composition and whether suppression (method for reducing background conductivity of mobile phase) technology is employed. The magnitude of the change is compared to a set of calibration standards and the concentrations quantified based on that comparison.

##### Amperometric

Amperometric detection is based on the presence of functional groups in the analyte that are readily oxidized or reduced. In this type of detector a voltage is applied between a working electrode and a reference electrode. When the target analyte, whose half-wave potential is such that the applied voltage causes a reduction or oxidation of the functional group(s), passes between the electrodes a current will flow [124]. This current is measured and compared to a calibration standard for quantification.

##### Spectroscopic Detection Methods

The detection of transition metal nutrient ions (e.g., Fe^2+/3+^, Mn^2+^) is most commonly achieved using UV/visible absorption following a post-separation reaction, although conductivity detection can be used in unsuppressed systems [124]. Complexing agents are added to the mobile phase (improves separation) and the sample is carried to the stationary phase where the transition metal ions are separated, typically via ion-exchange. The separated ions are then mixed with a chromophoric complexing agent that displaces the complexing agent previous added [124]. The absorbance of the newly formed UV- or visible-absorbing complexes is measured and quantified against calibrated standards.

#### 3.1.3. Suppression

Some IC methods employ suppression technology. The basic idea of suppressors is to chemically reduce the high background conductivity of electrolytes used in some mobile phase solutions [122]. The suppression process also serves to convert the analyte ions into more conductive forms, thereby improving resolution [122].

**Figure 5.**
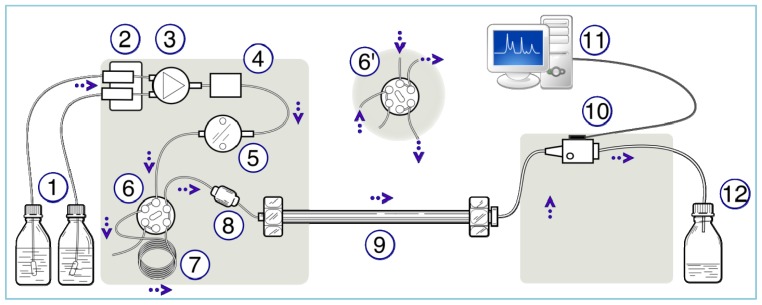
Basic components and flow paths in a conventional HPLC/IC system. (1) Solvent reservoirs, (2) Solvent degasser, (3) Gradient valve, (4) Mixing vessel for delivery of the mobile phase, (5) High-pressure pump, (6) Switching valve in “sample inject position”, (6′) Switching valve in “sample load position”, (7) Sample injection loop (10–100 μL), (8) Pre-column (guard column), (9) Analytical column, (10) Detector (*i.e.*, IR, UV), (11) Data acquisition, (12) Waste or fraction collector. Image used under the GNU Free Documentation License; Source file available at: http://upload.wikimedia.org/wikipedia/commons/thumb/a/a0/HPLC_apparatus.svg/2000px-HPLC_apparatus.svg.png.

Chromatography is the collective term for the physical-chemical separation of an analyte between a liquid or *mobile* phase and a *stationary* phase [122]. A generalized schematic of an HPLC/IC system is detailed in Figure 5 and briefly described here [122]. The mobile phase is degassed and then delivered to the system via a high-pressure piston pump. The sample containing the analyte(s) of interest is loaded into a sample loop (typical loop volume between 10–100 μL) via manual or automated sample loading systems. An injector valve temporarily puts the sample loop in-line with the mobile phase and the sample is carried to the separator column (stationary phase) where the analytes are temporarily retained. As additional mobile phase passes through the stationary phase, the analyte ions are re-mobilized at different rates according the ion-exchange characteristics of the system. The analyte(s) are then carried to a detector, which integrates the response and compares it to a stored calibrated response.

Ion chromatography is considered the ground-based, off-line standard for nutrient solution chemical analysis [125]. A typical greenhouse will obtain detailed reports of their nutrient solution by sending samples of their hydroponic solutions to accredited laboratories that conduct HPLC analysis and provide growers precise, multi-component analysis reports.

Conventional HPLC devices typically have relatively high mass, volume and power requirements [126]. In addition, HPLC systems are relatively complex, require substantial maintenance, toxic chemicals (depending on analysis), and have significant consumable requirements (e.g., solvents, columns, sample vials). Even with their high flexibility and precision, the aforementioned characteristics leave them not well positioned for spaceflight [127]. In particular, even an HPLC-on-chip, which diminishes the mass and volume issues, does not rank well in comparison to other potential technologies for portable diagnostic systems considered for future space exploration missions [128]. In particular, separation-based technologies fared less well compared to other assessed technologies due to greater crew time, maintenance and sample preparation requirements to name a few.

### 3.2. Ion-Selective Electrodes (ISEs)

An ion-selective electrode (ISE) utilizes the ion-selective properties of specialized materials to generate an electrical signal that can be measured. In the case of ISEs, the behaviour of ion-selective materials is due to ion-selective components that are included in their design. Their general functioning and development have been described in several key reviews [18,129–134]. Modern ISEs typically make use of a polymer membrane embedded with an ionophore that selectively binds a target ion of interest, along with an anionic site that serves to balance charge within the polymer matrix. The function of an ISE is similar to that of a standard pH probe, which is a selective electrode for H^+^ ions. For the pH probe, a glass bulb sensitive to H^+^ acts as the selective material that separates the sensor and the measurement environment. For other ISEs the material selected for the ion-sensitive surface will vary depending on the target ion.

**Figure 6.**
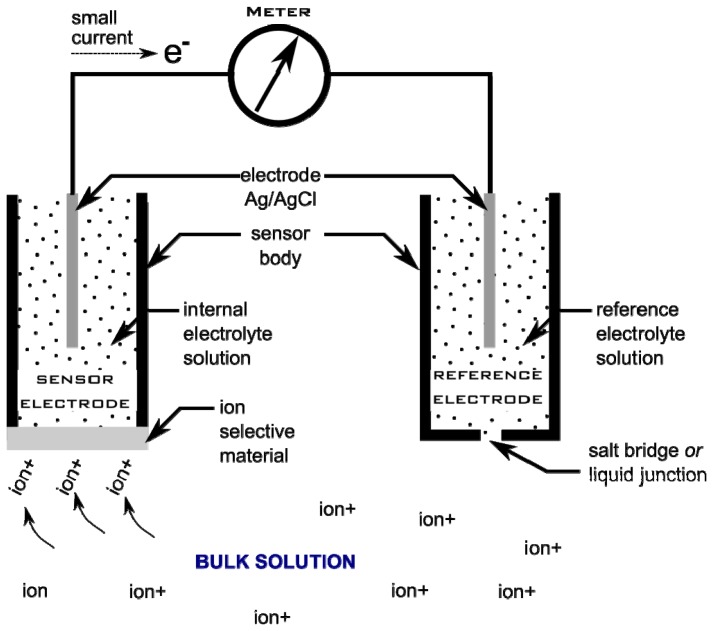
Illustration of the standard components of an ISE system used for activity measurements in solution.

The electrode works by comparing the electrical potential measured at the solution/sensor interface with that measured at a reference electrode. The sensor/reference relationship is shown schematically in Figure 6. When the sensor system (*i.e.*, the sensor electrode and the reference electrode combined) is placed into a bulk solution containing target ions, the ion-selective components will bind the target ions until an electrochemical equilibrium is reached. At this point, a charge develops at the interface between the ion-selective material and the internal electrolyte solution of the sensor electrode. It is important to note that the charge is proportional to the amount of ionophore (ion-selective component) that has bound the target ion. Connecting the sensor electrode with the reference electrode creates a simple galvanic cell capable of driving electrons from one node to another. This small signal can be measured using a sensitive meter. As the electrical potential at the reference electrode is relatively constant and the sensor responds selectively to target ions, the electrical potential at the solution/sensor interface can be correlated to the activity of the analyte in the bulk solution.

Modern electrodes are typically “combined electrodes” meaning that the sensor and reference electrodes are housed within one sensor body and many specialized styles exist.

ISEs have seen a great deal of attention over the past few decades and have been applied to a wide suite of applications. There has been great interest in their specific relevance in soil and nutrient solution monitoring for plant growth [5,6,36,135–138]. And there have been several explicit tests of ISEs for hydroponic nutrient solution monitoring [5,14,41,42,44,139,140]. Concluding remarks included; “The ion selective electrodes used here proved to be temperamental devices with a number of inherent problems.” [139], “In light of the potential limitations of ISE technology in individual ion based control systems, other advanced sensor types are being explored.” [14], “Yet, a number of practical difficulties still need to be resolved, in particular the stability and robustness of the measuring system, and the life expectation of the sensors.” [42], “No sensors of acceptable quality have been offered on the market yet.” [44]. The literature raises the following specific limitations with ISEs, drift/stability [5,14,44,140–143], electrical interference [144–148], temperature sensitivity [14,140,142], lifetime [42,44] and the requirement of a reference electrode [144].

If considered for use in a recirculating nutrient control system for space-based systems, the above issues would result in certain operational constraints. In particular, to remedy the drift/stability issue with ISEs a regular calibration sequence should be implemented [5]. This regular calibration program could necessitate a considerable demand on crew time (depending on automation) but would prevent system closure if discarding the used calibration solutions, or could result in ion accumulation if considering the reincorporation of the used calibration solutions back into the nutrient system [14].

That said, other work suggested that ion-selective electrodes used in conjunction with other sensor technology could serve as a useful strategy for space-based life support systems [41]. This work and associated work tested measured nitrate and chloride concentrations using ion-selective electrodes [41,85]. ISEs now exist for a wide-range of analytes and are offered by numerous traditional laboratory supply companies. Single ISEs incorporating sensing membranes for a number of different analytes are also now available from commercial providers and continue to improve over more conventional electrodes [149] and new generation ISEs will likely continue to overcome the aforementioned limitations.

### 3.3. Ion-Selective Field Effect Transistors (ISFETs)

**Figure 7.**
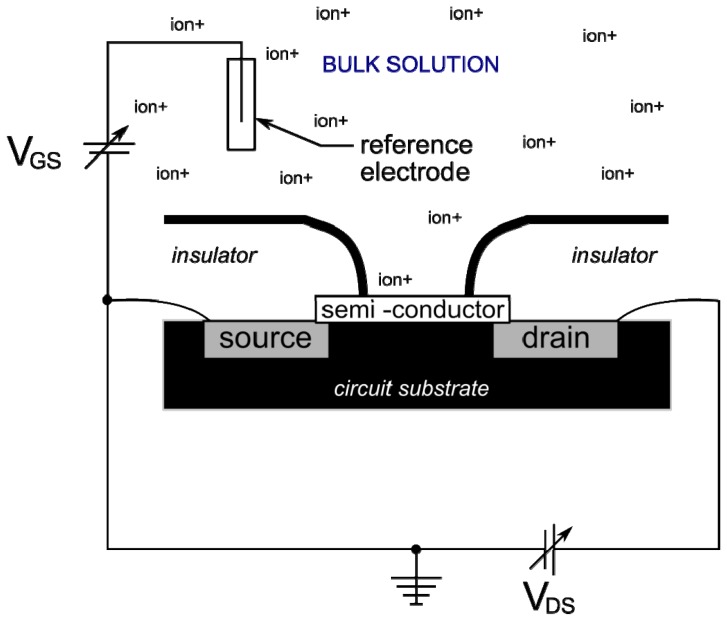
Illustration of the standard components of an ISFET used for ion activity measurement in solution.

Ion-selective field effect transistors (ISFETs) which incorporate much of the same technology as ISEs, have been discussed in significant detail in several general reviews [150–153]. ISFETs also take advantage of the properties of specialized materials that have been designed to respond to the activity of a target ion in solution. As the name would imply, ISFETs use a small transistor circuit, a common electrical device composed of a metal-oxide semi-conductor that connects two metal contacts (termed the source and the drain). The device is shown schematically in Figure 7. Due to the unique properties of semi-conductors, there exists a threshold voltage for which the semi-conductor will generate electron channels, switch its function from an insulator to a conductor, and allow a small current to flow through. The key features of an ISFET are that the threshold voltage required to close the circuit is a function of ion activity within the solution and specialized semi-conductors can be used for selectivity to particular ions. Referring to Figure 6 below, the gate-voltage (V_GS_—varies with ion activity) of an ISFET is measured at a reference electrode in contact with the bulk solution, while the drain-voltage (V_DS_—remains relatively constant) is measured at the drain. By comparing the gate-voltage with the drain-voltage the sensor response can be related to analyte activity.

Like ISEs there has also been substantial interest in the application of ISFETs to soil and hydroponic growth systems [49,142,154,155]. Several other references include discussion of both ISE and ISFET sensor types with relevance to plant growth systems [42,44,141,156,157]. Compared to ISEs, ISFETs allow for further miniaturization, reduced response times and reduced susceptibility to external electromagnetic fields [154]. Besides these benefits, ISFETs face many of the limitations of ISEs, have only witnessed very limited incremental changes since their introduction more than 30 years ago and have yet to find solid commercial footing [153]. A survey of published limitations includes the following, drift /stability [44,133,142,155,158], temperature sensitivity [142,155], lifetime [44] and the requirement for a reference electrode [151,153,155]. Due to their small relative size, ISFETs require less calibration solution than ISEs which would reduce the issues related to discarding or reintroducing these solutions back into the nutrient solution [142].

### 3.4. Absorption/Atomic Spectroscopy (Abs-Atm)

Direct absorption spectroscopy whereby light is directed through a sample solution and the incident light is related to the output light by way of Beer's Law can be used to estimate the concentration of a given component within a sample. Unfortunately, not all analytes of interest (especially inorganic compounds) interact with light and can thus be measured using such direct, or ‘primary absorption’ based techniques. Fortunately, certain metals such as iron, manganese, copper, zinc have a tendency to form large complexes in aqueous solutions and subsequently absorb strongly in the UV-Vis region. The direct absorption of these coordination compounds can then be utilized to estimate the activities of these transition metals.

**Figure 8.**
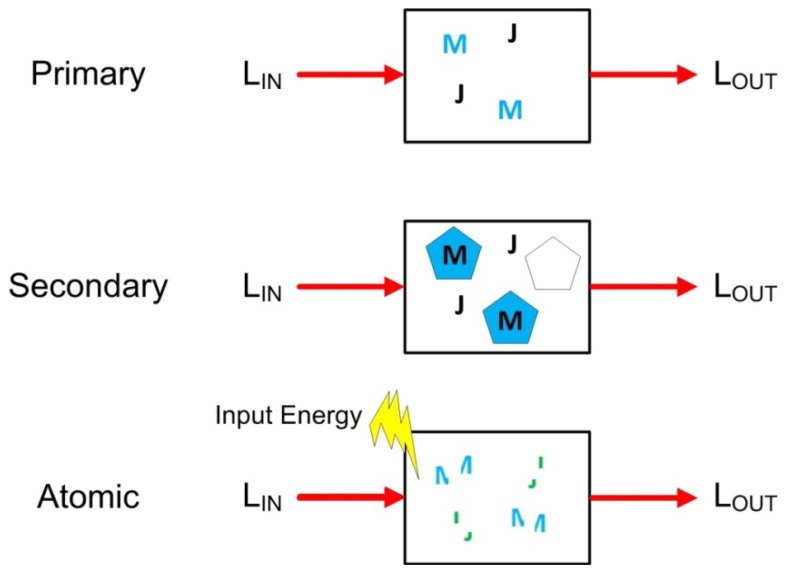
Simplified representation of three forms of considered absorption and atomic absorption techniques. Primary absorption involves direct absorption spectroscopy of the sample. Secondary absorption involves the addition of chromogenic compounds that permit the selective measure of certain ions. Atomic absorption spectroscopy uses different forms of input energy to obtain the atomic form of the contained components that have characteristic optical properties. L_IN_ = incident light, L_OUT_ = transmitted light, M = analyte of interest, J = interfering species, = chromogenic complexing agent.

Only a limited number of ions directly absorb in an accessible water transmission band or form large coordination compounds in aqueous solutions which consequently absorb light. To resolve those which do not, a ‘secondary absorption’ method has been developed in which chromogenic compounds can be added to the solution that interact selectively with the analyte of interest and thus be utilized as an optical tool to quantitatively measure the analyte. The disadvantage of this technique is that it requires the addition of these chromogenic compounds. The basic principles of primary and secondary absorption spectroscopy are graphically shown in Figure 8.

Atomic spectroscopy is an additional technique in which energy is applied to break the molecular bonds of a sample and generate atomic elements which all absorb or emit at characteristic accessible wavelengths, thus permitting quantitative assessment. Various forms of atomic spectroscopy exist depending on the form of source energy used to break the molecular bonds (e.g., flame, electrothermal, electric arc, inductive coupling, laser, *etc.*) and if the measurement is absorption or emission based, *etc.* Atomic absorption spectroscopy is also graphically illustrated in Figure 5.

It is important to note that in complex solutions such as hydroponic nutrient solution that all three of the described sensing methods experience the challenge that absorption at any one wavelength can be due to multiple absorbing species within the solution. This fact may complicate data analysis and require the use of pattern recognition algorithms to isolate individual species.

NASA has investigated direct absorption spectrometry and liquid atomic emission spectrometry (electric arc induced) of nutrient solution samples through their Small Business Innovation Research (SBIR) program [121,159,160]. Atomic spectrometry has been used separately or in tandem with absorption spectroscopy, and although these systems are decreasing in mass, power and volume with time, they typically require larger, more bench-top type sized instruments [41,161]. Conversely, laser induced breakdown spectroscopy (a form of atomic emission spectroscopy) has shown good progress as a low-mass, in-situ measurement tool and has more recently been applied to monitoring within agricultural solutions [162,163].

Unfortunately, certain systems such as the liquid atomic emission spectrometry and inductively coupled plasma (ICP) spectroscopy hardware proposed for space plant growth systems cannot be used to monitor all of the nutrient ions of interest [41,121,159,160]. For example, ICP spectroscopy cannot be used to assay for nitrogen, for which knowledge will be critical for hydroponic nutrient solution monitoring [41]. Primary absorption spectroscopy is also limited, lacking the capacity to monitor for potassium, calcium, magnesium and several other critical nutrient ions [121,160]. As direct inorganic measurement of nutrient samples cannot presently measure all ions, other selective optical sensing techniques have been explored.

### 3.5. Colorimetric Solid Phase Extraction (CSPE)

Solid phase extraction is a laboratory technique commonly utilized as a separation process to separate a component of interest dissolved or suspend in a liquid, from other components. It can thus be utilized to isolate/concentrate an analyte of interest from a wide number of samples (blood, urine, water, *etc.*). Solid phase extraction includes a ‘solid phase’ (solid sorbent) into which the component of interest is extracted (or which instead extracts all of the sample impurities except the analyte) and exhibits a number of advantages over liquid/liquid extraction including improved yields, improved speed and reduced requirements for the use and disposal of organic solvents [164]. Depending on the choice of the solid and liquid phases, there are a number of types of solid phase extraction mechanisms (normal phase, reverse phase, adsorption, *etc.*). Although other arrangements are possible, typical solid phase extraction detection systems utilize a syringe to push a liquid sample through a specially designed cartridge holding the solid phase.

**Figure 9.**
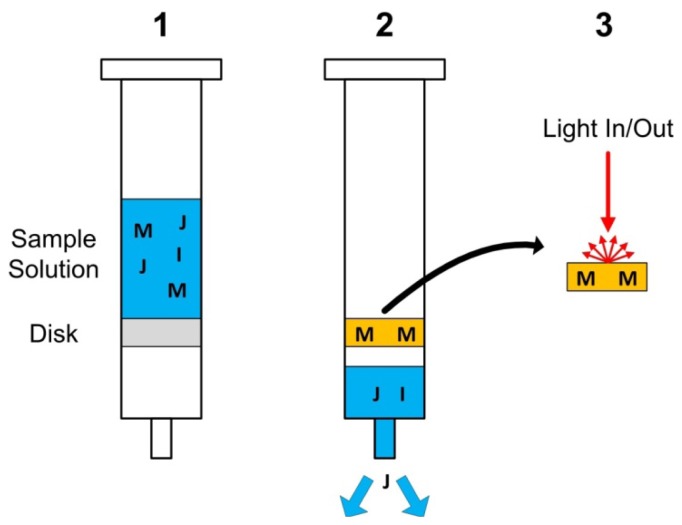
Simplified representation of the basic operational principles of colorimetric solid phase extraction. The sample solution of interest including impurities (I,J) is passed through a disk made up of an appropriate solid phase which extracts the analyte (M). Following pass through, the single-use disk is interrogated by light and the change in its diffuse reflection spectra can be related to the analyte ion activity.

When solid phase extraction is combined with a matrix material that undergoes a change in its optical properties based upon the uptake of the analyte of interest, it is termed colorimetric solid phase extraction (CSPE). A graphical representation of CSPE is presented in Figure 9. This arrangement is useful as it permits relatively rapid analysis (*i.e.*, of the solid phase itself) in comparison to the additional steps that are normally included in the solid phase extraction process. In particular, an elution step is often utilized following extraction to recapture the analyte from the solid phase. Nominally, elution is achieved by pushing clean solvent (eluent) through the disk and results in the output of a sufficiently concentrated output solution to permit detection by a separate detection scheme. Alternatively, CSPE directly measures the optical changes of the solid phase due to a contained colorimetric reagent [165]. This impregnation of the solid phase by a colorimetric reagent allows analyte to be concurrently complexed upon extraction, driving a colour change. The amount of colour change (typically achieved through analysis of diffuse reflection spectra) can be related to the analyte ion concentration. Diffuse reflectance spectroscopy monitors the radiation reflected from a rough/textured surface such as a colorimetric solid phase extraction membrane [165]. In the instance of textured surfaces, most of the reflected light is diffusely reflected (cf. specularly reflected). Diffuse reflectance spectra can then be analyzed through Kubelka-Munk theory in which the ratio of incident and reflected light intensities can be utilized in an analogous manner to Beer's Law for absorbance spectra [166].

While solid phase extraction could be considered to be more of a laboratory based technique, CSPE allows measurements to be more easily made in-situ. Both techniques can provide powerful solutions but must consider the careful selection of both the utilized solvents and solid sorbents (as options are diverse) for a given analyte in question. Sorbent materials in solid phase extraction are of similar nature to those utilized in HPLC columns and most often are based upon silica (bonded to a specific functional group) as the support material as silica has the advantage of being available in a wide range of well-defined surface areas and pore sizes while being of relatively low cost [167]. Solid phase extraction disks are classically designed for single-use and thus a new disk is utilized for each analyte measurement. Further developments are likely in the area of miniaturization and on-line application of solid phase extraction based sensors [167].

CSPE techniques have been developed to measure water contaminants at very low detection limits and similar techniques could be applied nutrient ions of interest [165]. The primarily disadvantage of present CSPE technologies are that they are single-use and thus would not easily be deployable into on-line nutrient solution or water monitoring systems.

### 3.6. Ion-Selective Optrodes

In general, optrodes (the term “optode” is also used and is analogous to “optrode”) exhibit some change in optical properties in a manner that is selective to an analyte of interest. Typically this is achieved through the selective complexation of the analyte with an indicator dye (*i.e.*, chromoionophore), a critical component of an optrode. Although no formally presented classification of optrode types exist, they can be classified based on the following top-level characteristics:

- Surface or bulk mechanism
- Reversible or irreversible
- Optical transduction scheme (absorption, fluorescence, *etc.*)

**Figure 10.**
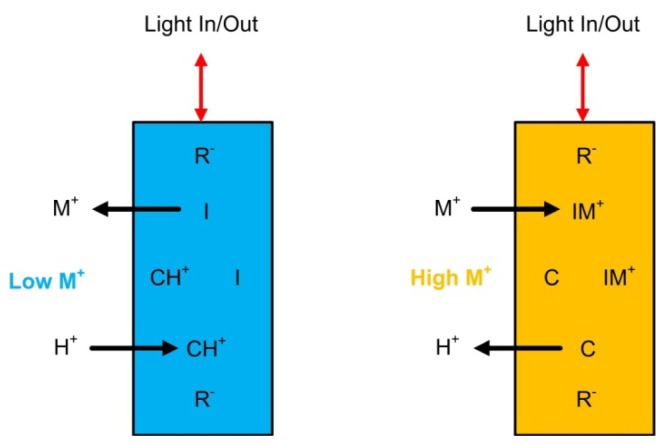
Summary of the ion-exchange sensing mechanism that forms the basis of bulk optrode functionality. When exposed to low analyte ion (M^+^) activity the optrode membrane is one colour (left) while when exposed to high analyte ion activity the film undergoes a change in its optical properties and appears a different colour (right). This colour change is typically sensed through an attached spectrometer. The ion-exchange mechanism involves the exchange of the analyte ion and the hydrogen ion into and out of the film; C = chromoionophore, I = ionophore, R^−^ = anionic site, IM^+^ = ionophore-analyte ion complex, CH^+^ = chromoionophore-hydrogen ion complex.

Surface optrodes rely on surface phenomena whereby the active components of the optrode are immobilized near the interface or surface of the optical element and are thus located within the sample solution [168–170]. Bulk optrodes on the other hand generate an optical response based upon the concentration change of some species within a separate, membrane phase (*i.e.*, driven by the activity of the target ion within the sample). The active components of bulk optrodes are uniformly distributed within this separate, bulk phase while the active components of a surface optrode are situated solely at the optrode and sample solution interface [170]. Further specifics on the difference in surface and bulk optrodes have been described elsewhere [171]. Figure 10 illustrates the general operational principles of bulk optrodes. Their function is based upon an ion-exchange mechanism in which ions are exchanged into and out of the optrode membranes. This exchange results in changes in the level of complexation of a coloured molecule (chromoionophore) and thus changes the optical properties of the membrane itself. These optical changes can be sensed by a spectrometer or some other light detection system.

Optrode membranes are typically of highly plasticized polymer construction and incorporate two, but more often three active membrane components:

- Ionophore: a molecule that selectively binds to the analyte ion of interest and can aid in its transport into the membrane.
- Chromoionophore: a molecule that selectively binds to a particular ion and undergoes a change in one or more of its optical properties based upon this binding.
- Ionic sites: charged molecules added to the membrane and associated with membrane electroneutrality, their concentration influences the amount of exchangeable ions of opposite charge permitted within the film.

Membrane components can be changed depending upon the analyte or ion of interest and their concentrations varied to influence the dynamic range of the sensor and other sensor parameters. Membrane components can be covalently immobilized or physically entrapped within the membrane. The absorption, fluorescence or other optical properties of an optrode membrane can be monitored using a number of different hardware arrangements (membrane placed at the end of an optical fibre, evanescent wave sensing, *etc.*).

The application of optrodes to nutrient solution monitoring was first explored in the mid-1990s [172,173]. Specifically this research group focused development work on controlled environment optrodes for pH, potassium, calcium, magnesium, aromatic hydrocarbons, ammonia sensors (vapour and dissolved) as well as atmospheric constituents including carbon dioxide, moisture, ethylene, carbon monoxide and hydrazine [120,172–175]. It should be noted that the potassium, calcium and magnesium sensor research was focused on optimizing molecular probe design and less on fully integrated sensor designs which would be necessary for monitoring nutrient solution within an operational plant growth system. The bulk of this prior work was conducted under the NASA SBIR Program by Polestar Technologies Inc. and GEO-CENTERS Inc. but did not result in sensors which have widespread use today [176]. It is relevant to note that work in this area did not stop due to issues associated with technical feasibility but instead, programmatic motives drove the research away from optical ion-selective monitoring and toward a focus on optical bacteria monitoring [176].

Recent work in the area of bulk optrode sensors (general reviews [18,177]) with application to plant growth systems has also been conducted [116,171,178]. Sensors have been shown to demonstrate the selectivities required for measurement of hydroponic solutions but have been shown to exhibit limited lifetimes, although several operational workarounds have been proposed [116,171].

### 3.7. Comparison of Ion-Selective Sensing Technologies

The development of reliable sensors for ion-selective sensing of nutrient solution has proven to be a difficult challenge with current sensor technology having insufficient robustness and/or relatively high equivalent system mass, limiting both their use terrestrially and feasibility for use in space-based life support systems [16].

Table 2 presents a summary assessment of present ion-selective sensor technologies and a general evaluation of parameters that could be considered important in selection. The first important distinction between sensing systems is if they provide single or multi-component detection. As evident from Table 2, HPLC and absorption/atomic spectroscopy are typically multi-component detection schemes (although some exceptions exist), while ISE, ISFET, CSPE and optrode technologies are typically single-ion detection systems. With design improvements and miniaturization, several of the single-ion detection technologies are being designed to act as sensors for more than one analyte (e.g., several ion-selective membranes incorporated into a single ISE), but it is important to note that at the fundamental level, sensors still require a specific sensing element for each analyte. The single *versus* multi-component sensing distinction is an important consideration to how each sensor technology was evaluated for each metric presented in Table 2. For example, from the perspective of mass, power and volume, although HPLC systems are typically heavier, use more power and more volume, they provide multi-component information and thus depending on system requirements, may still provide a more appropriate solution than utilizing multiple single-component detection schemes in tandem.

Other evaluation metrics include if technologies can be used in an on-line manner to provide for more automated and real-time sensing information or if sensing is conducted off-line, which often requires user collected sampling, sample processing and a longer delay in the attainment of results. Table 2 also presents comparative parameters such as cost, measurement type, accuracy, calibration/consumable requirements, training requirements, hazards; sensor mass, power and volume; and finally, two different metrics for how established or ‘ready’ the technology is for use. Cost factors in the overall infrastructure and hardware costs associated with acquiring, operating and maintaining an ion-selective sensing system. The fundamental measurement category provides a distinction between those sensors that measure ion activity and those that measure concentration. Accuracy includes considerations with respect to how selective a given sensor is for the analyte(s) in question and the general confidence level of a given measurement (e.g., presently more accuracy can be achieved with an HPLC than it can with an optrode). Calibration/consumable requirements include considerations with respect to the required time to setup for a given measurements sequence as well as the consumables that may be required to conduct a measurement or that are changed out after a fixed period of operation (e.g., calibration solutions, columns, films, *etc.*). Training requirements include the general expertise required by system operators and the amount of training and time required to ensure appropriate use and maintenance of a given instrument. Hazards include instrument requirements for toxic chemicals, high voltages, high powered lasers or any other consideration that could present a safety risk. The authors’ have also suggested an evaluation on how proven the listed technologies are for terrestrial applications with the ‘established technology’ metric and with the technology readiness level (TRL) metric for space-related applications. TRL is an often utilized metric that is an important consideration of the selection of hardware for flight. This metric incorporates how “space-ready” hardware is and if a given technology has any flight heritage, or if it has been tested in a relevant environment or under relevant conditions [179].

Although assessment of the most appropriate technology for application terrestrially and for application in space systems requires consideration of many of the same parameters, several important differences exist. For example, the mass, volume and power of a given technology are considerably more important in space-based applications than they are on the ground, where although important, they are usually not critical in selection. For instance, in greenhouses, the power of a sensing system is typically far less than other operational components (e.g., pumps, fans) and thus is not an important factor in selection. In space where resources are more constrained, the mass, volume and power typically drive the technology trade study more than any other consideration.

Hazards, although always an important technology selection consideration, must be even more exhaustively considered for space systems. Chemical use on-orbit is highly controlled, requiring comprehensive safety reviews and containment strategies. Given this, chemicals should be eliminated to the greatest extent possible considering the closed-environment of the space vehicles [180,181]. Those ion-selective sensor systems utilizing potentially hazardous materials will be penalized when conducting on-orbit sensor trade-studies while they may be more appropriate in terrestrial systems. The on-line and calibration/consumable metrics are additional distinctions, as space-based systems should incorporate substantial automation to minimize crew time as well as include consideration of the solutions and other consumables that may be required in the calibration and use of a given system. These components must be included in launch manifests and must then be rightly considered in disposal and waste management strategies, whereas on the ground these are less of a concern from a logistics perspective.

Table 2 includes parameters relevant to both terrestrial and space-based technology trade-studies. The weighting factors for these different parameters could be adjusted depending on the operational environment. The rough assessment parameters provided in Table 1 are based upon the authors’ assessment of the literature and experimental experience with some of the ion-selective technologies and are meant to serve as a top-level comparative tool.

### 3.8. Overall Sensing Systems Requirements

As described, although there are approximately 15 to 18 nutrient elements required by plants, certain of these elements can exist in several forms. Two key examples include nitrogen and phosphorus which can exist in nutrient solution as ammonium (NH_4_^+^), nitrate (NO_3_^−^) and monohydrogen phosphate (HPO_4_^2−^), dihydrogen phosphate (H_2_PO_4_^−^) respectively. In theory then, for a comprehensive understanding of the hydroponic solution, it is not the knowledge of only 15 to 18 species that is required, but knowledge of all the nutrient ions/forms within solution. Further, certain non-essential nutrients, such as sodium (although occasionally considered essential for select higher plants) due to their high levels in some source water, should also be considered as important candidates for ion-selective monitoring. In practise, reliable sensors for several ions would provide a considerable step forward from the current state of the art. Presently none of the described sensor systems provide an ideal solution for either terrestrial or space-based plant growth systems. All sensor types require further development work, especially when considering the additional requirements that space-based systems require.

In August 2003, NASA organized an expert panel which reviewed submittals for several potential systems for water quality monitoring on-board the ISS [182]. Although the review process only included technologies for which organizations submitted as a response to the NASA request for information, the results serve as an additional reference for the criteria for how different ion-selective technologies for space-based nutrient solution monitoring could be evaluated. These criteria included; operation in a spacecraft environment, instrument characteristics, system characteristics, compounds and instrument maintainability. The technologies were considered both from their presently ‘demonstrated’ performance parameters as well as their ‘potential’ performance parameters, should they be further developed. Furthermore, although this study included systems for both organic and inorganic monitoring, those technologies for inorganic monitoring included systems based upon ion chromatography, electrodes/voltammetry and UV-Vis absorption spectrometry. Several important conclusions were drawn related to the inorganic sensor types described above. In the short to mid-term the expert panel reached the consensus that CSPE, even with its disadvantages, was the only one of the considered approaches that could be reasonably implemented. No consensus could be reached for the most appropriate mid to long-term inorganic sensing technology.

Table 2 incorporates the relevant findings of the described study and provides a useful starting point for assessing ion-selective sensing technologies and priority development areas for each respective technology for either terrestrial or space-based plant production systems. It is obvious that more work is required to further develop all of the described ion-selective sensing technologies. Additional characterization information is required to more ably conduct improved trade studies. Furthermore, additional plant growth system integrated studies are required to enhance the understanding of sensor performance under realistic operational environments/protocols.

## 4. Other Space-Based Ion-Selective Sensor Applications

In addition to the monitoring of space-based plant growth systems, there are several current and potential applications for ion-selective sensors for use either on-orbit or on planetary exploration missions. These additional applications could utilize sensors for some of the same ions as required for plant growth systems but also introduce the requirement to measure other ions. Several past and present applications and specific development efforts are described in the sections to follow.

The Portable Clinical Blood Analyzer (PCBA) is a device based upon the commercial i-STAT device which strives for point-of-care blood testing and has been tested in Aquarius, the underwater space analogue facility; has flown aboard the Space Shuttle and is presently on board the ISS [183–186]. The PCBA tests blood samples for a number of parameters and includes ISE based techniques for the testing of sodium, potassium and calcium [185]. The PCBA is a compact, portable instrument measuring 3.5 cm × 6.5 cm × 18 cm with a mass of 0.54 kg and can process small microliter liquid samples in approximately 120 s [183]. In addition to being available for crew medical operations on the ISS, the instrument also supports science experiments which benefit from in-situ constituent reports of liquid samples [183].

ISE technology has also been flown aboard the Mars Phoenix Lander which landed in the northern plains of Mars in 2008 [187]. Phoenix's Mars Environmental Compatibility Assessment (MECA) Instrument included four separate single-use Wet Chemistry Laboratory (WCL) cells. Each of these cells contained hardware for the conduct of aqueous chemical analysis of the soil using, among other sensors, a suite of ISEs [127,188]. Activities of a number of cations (Ca^2+^, Mg^2+^, K^+^, NH_4_^+^, Na^+^, H^+^) halide ions (Cl^–^, Br^–^, and I^–^) and several others were measured in-situ [127,187]. Work demonstrated that the polymer-based ISEs proved to be more resilient than initially thought as they were able to survive a set of relatively harsh conditions such as heat and humidity on the launch pad at Kennedy Space Center, desiccating vacuum during transit and extreme temperatures on the surface of Mars [188]. The nature of the integrated sensor array in which a wide slate of ions were measured, permitted further conclusions to be elucidated regarding the analyzed samples and assisted in the removal of certain errors or uncertainties in given electrodes [187,189]. Several of the same researchers have proposed a similar device for the measurement of on-orbit water quality on the ISS [190,191].

### On-Orbit Water Quality Monitoring

Until Skylab no in-flight water monitoring technologies had been incorporated into US spacecraft [192,193]. Although far from ideal, this was primarily possible due to the relatively short duration missions and lack of requirement for re-use of the launched water. Source water for these flights (save fuel cell generated water) was collected from a source of appropriate quality on the ground; nominally tested and a disinfectant often added. For example the Mercury program used water as collected from the public water system in Cocoa Beach, Florida, while later programs added biocides to the source water either before and/or after launch [194].

For longer duration flights, it is not possible to assume that even stored water quality will not change over time and thus the importance of in-flight monitoring becomes more critical. For example, although Skylab did not incorporate any water recycling and instead just utilized stowed water, a sampler was utilized to assess iodine biocide levels by fixing samples with a linear starch reagent and subsequently comparing them to photographic standards [195–197]. As water recovery systems are implemented, there is the potential for further variation in on-orbit water quality and further suggests the need for more detailed water monitoring. In the 1990s NASA asked the National Research Council to develop guidelines on exposure to contaminants in spacecraft water. This effort resulted in several key documents defining those chemical contaminants (not microbial agents) which may adversely affect space crews and thus which should be considered in active water quality campaigns [198–201]. There have been numerous reported water quality issues on-orbit in the Shuttle, Mir and more recently aboard the ISS. Selected examples have included high iodine levels in Shuttle water, caprolactam in stored water in contingency water containers (CWC) on the ISS and ethylene glycol coolant leaks from the thermal control system aboard Mir which subsequently resulted in high levels within the collected humidity condensate [198,202,203].

The current approach with ISS water quality monitoring is primarily off-line in nature, whereby water samples are collected on-orbit and transferred to the ground for detailed analysis on returning vehicles. Although ground analysis permits full chemical and microbial characterization (>250 analytes [202]), this approach faces a number of challenges including; infrequent and irregular return and analysis frequency, limited return water volumes and potential degradation of samples during storage [204,205]. In particular, due to the lag between obtaining water quality information (on the order of months [206]), the potential of a water quality anomaly (such as those described) resulting in crew risk is significantly increased. These challenges could be alleviated with the implementation of more improved on-line monitoring capabilities in-flight.

**Figure 11.**
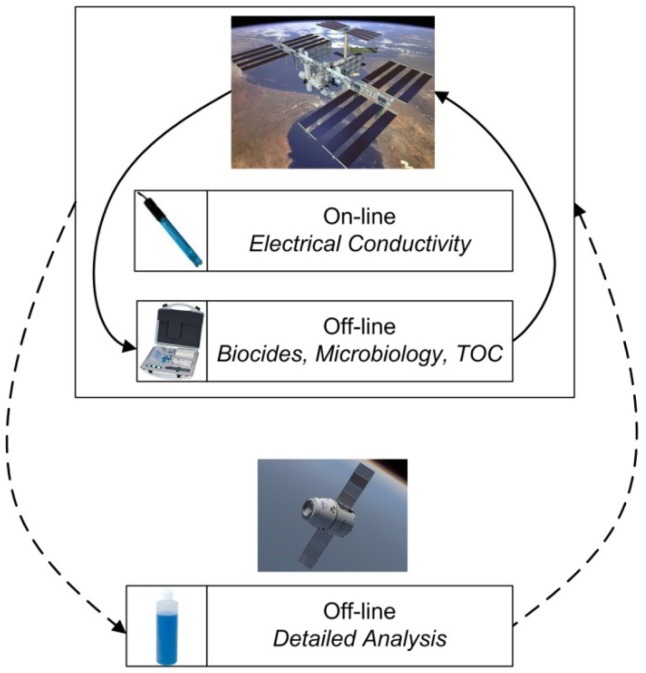
Current ISS water monitoring capabilities (US Orbital Segment). On-line monitoring of EC exists within the Water Processor Assembly. TOC analysis can be conducted in an on-line manner through the TOCA directly piped into the water system and conducted on an approximately weekly basis. Other off-line analyses can be conducted on-orbit utilizing water test-kits for detection of biocides and microbiology. Detailed analysis is also conducted on an infrequent basis with ground returned samples.

Even with on-orbit water processing systems, in-flight monitoring capability has been extremely limited. Mir included conductivity sensing but this was the only process control monitoring that was conducted on-line [198,203]. Although there were initially considerable plans for in-flight water quality monitoring on-board the ISS, such as an on-line requirement for the monitoring of conductivity, pH, temperature, iodine and total organic carbon (TOC); only on-line conductivity sensing has been implemented [207]. Conductivity measurements are being conducted in-line within the ISS Water Recovery System [208]. TOC is also analyzed in-flight, but in an off-line manner through the use of the Total Organic Carbon Analyzer (TOCA) that is plumbed into the Water Processing Assembly product line and which the crew can initiate an automatic analysis procedure by way of the TOCA interface screen [209]. Figure 11 presents a summary of the present on-line and off-line water quality monitoring capabilities on-board the ISS.

Of particular interest for on-orbit ion-selective monitoring are the utilized biocides, added to limit microbial growth and biofilm formation within spacecraft water systems [198]. For example, while silver is utilized on the Russian segment of the ISS, the US segment uses iodine, both of which are not presently monitored in an on-line fashion [166]. In 2009 an experimental ion-selective detection scheme utilizing CSPE was tested aboard the ISS for near real-time detection of molecular iodine and silver and the same techniques have been previously explored for the detection of other ions [165,166,205,210,211]. This developed Colorimetric Water Quality Monitoring Kit was certified in 2011 and is now considered operational hardware on the ISS [204].

On-line ion-selective calcium monitoring is also of immediate interest due to the increased calcium levels excreted in astronaut urine (in the microgravity environment) and its facility in forming precipitates that damage and block water treatment hardware, a critical reported issue with the ISS Urine Processing Assembly [212]. A low mass, low power and no consumable multi-analyte measurement system for water quality measurement has been denoted as a priority technology item in recent NASA roadmapping activities, providing further evidence of their benefit [213].

Other optrode type sensors have also been investigated for space applications including the commercial Paratrend multi-analyte sensor for on-orbit biomedical and experimental purposes. The Paratrend optical sensing system is capable of measuring pH, dissolved carbon dioxide, dissolved oxygen and temperature of a given sample [214]. In addition to NASA directed optrode research, it is also noteworthy, that around the same time, the European Space Agency (ESA) invested in basic optrode development for spaceflight as part of their Basic Technology Research Programme. The work involved optrode development for pH, oxygen and carbon dioxide and further demonstrates the merit of optrodes for space-based systems [215,216].

Other colorimetric techniques utilizing indicators (e.g., porphyrins) incorporated onto thin-films have also been proposed for inorganic on-orbit water monitoring [182,217]. These sensors can be interrogated in measurement systems in much the same way as the previously described bulk optrodes (light emitting diodes, fiber optics, mini-spectrometer) and thus require minimal mass, power and volume resources [218,219].

The comparison of present on-line *versus* off-line sensing capabilities within the ISS and in terrestrial greenhouses (Figures 8 and 2) is an interesting one. Both currently employ only limited on-line monitoring technologies but do obtain detailed water quality reports on an infrequent off-line basis and thus suffer limitations that would otherwise be avoided if more on-line monitoring was available.

Regolith/ice analysis, space-based water analysis and advanced biomedical diagnostic systems are just some of many potential applications where low mass, robust, *in situ*, ion-selective analysis technologies would be advantageous [127,128,220,221]. For missions to near Earth objects, the Moon or Mars, in-situ monitoring will become ever more critical due to the inaccessibility of Earth-based laboratory analyses.

## 5. Conclusions

Greenhouses presently utilize up to four methods to provide feedback for the adjustment of their hydroponic nutrient solutions. These methods include; (1) direct on-line sensing of the pH and EC of their nutrient solutions, (2) visual inspection of plants for nutrient deficiency or toxicity symptoms, (3) off-line analysis of plant leaf/tissue and (4) off-line laboratory analysis of hydroponic nutrient solutions. Although much of this information is still highly valuable and in some instances critical, on-line ion-selective nutrient solution sensors would provide considerable enhancement and underscore the reason that this technology has been so sought after by the greenhouse sector. On-line ion-selective sensing would increase crop yields, reliability, decrease water and nutrient requirements, aid growers in meeting ever tightening environmental regulations and provide considerable supplemental benefits. Ion-selective sensors are also required for the high yield, low water use plant production systems that will be implemented on future long duration space missions. Several different ion-selective sensing technologies have or could be applied for nutrient solution sensing including; high-performance liquid chromatography, direct absorption/atomic spectroscopy, ion-selective electrodes, ion-selective field effect transistors, colorimetric solid phase extraction and ion-selective optrodes. The same technologies are also being considered for on-orbit water quality monitoring and for other space related applications. Each technology is at a different level of readiness and harbors its own respective advantages and disadvantages. No technology yet provides an ideal solution for either terrestrial or space-based agriculture or water management systems and all sensor types presently warrant further investigation.

## Figures and Tables

**Table 1.** Water quality targets for raw irrigation water sources.

**Water Quality Parameter**	**Targets for Raw** ^a^ **Irrigation Source Water** ^b^
pH	5.8–6.0
Alkalinity	0.75–2.6 meq·L^−1^
Hardness	<150 mg CaCO_3_ L^−1^
Nitrate, Ammonium and Phosphorus	<5 mg·L^−1^ (higher indicates contamination)
Potassium	<10 mg·L^−1^ (higher indicates contamination)
Calcium	<100 mg·L^−1^
Magnesium	<50 mg·L^−1^
Sodium	<50 mg·L^−1^
Sulphate	<100 mg·L^−1^
Chloride	<100 mg·L^−1^
Iron	<5 mg·L^−1^
Boron	<0.5 mg·L^−1^
Copper	<0.2 mg·L^−1^
Fluoride	<1 mg·L^−1^

a Water that has not been modified for crop production (*i.e.*, has not had nutrients added, pH adjusted, *etc.*);

b Adapted from [72,76,77].

**Table 2.** Summary of ion-selective sensor technologies and their general performance for various considered technology metrics.

**Metric**	**HPLC**	**Abs-Atm**	**ISE**	**ISFET**	**CSPE**	**Optrode**
Component detection	Multi	Multi	Single	Single	Single	Single
On-line	Generally not	Most	Yes	Yes	No	Yes
Cost	High	Low-Med	Low-Med	Low	Low	Low
Fundamental measurement ^a^	Implementation dependent	Concentration	Activity	Activity	Activity	Activity
Accuracy	High	High	Med	Med	Med	Med
Calibration/consumable requirements	High	Low-Med	Med	Med	Low-Med ^b^	Low
Training requirements	Med-High	Low-Med	Low	Low	Low	Low
Hazards	Hazardouschemicals ^c^	Generally not ^d^	No	No	No	No
Established technology	High	Med-High	Med	Med	Low-Med	Low
Mass, power, volume	High	Low-High	Low	Low	Low	Low
Technology readiness level	Med	Med^d^	Med-High	Med	High	Low-Med

HPLC = High-Performance Liquid Chromatography, Abs-Atm = Absorption/Atomic Spectroscopy, ISE = Ion-Selective Electrode, ISFET = Ion-Selective Field Effect Transistor, CSPE = Colorimetric Solid Phase Extraction.

a Values are not necessarily categorical as there are certain instances when the measurement can depend on specific sensor designs. It should also be recalled that in ‘clean’, dilute (low ionic strength) sample solutions that activity and concentration are approximately equivalent.

b Current CSPE are single-use.

c Some methods.

d Some designs require high voltage power systems or lasers.

e Quite system dependent.
